# Fees for Notification of Infectious Diseases

**Published:** 1919-04-05

**Authors:** H. O. Stutchbury, B. A. Richmond

**Affiliations:** Secretary. The Association of Panel Committees. Staple House, 51 Chancery Lane, W.C. 2.


### THE EDITOR'S LETTER-BOX.

**[Correspondence on all subjects is invited, but we cannot in any way be responsible for the opinions expressed by our correspondents, who must give their name and address as a guarantee of good faith, but not necessarily for publication. Correspondents are reminded that brevity of style and conciseness of statement greatly facilitate early insertion.]**

### Fees for Notification of Infectious Diseases.

*To the Editor of* THE HOSPITAL.

SIR,—In reply to the representations made by the Association to the Local Government Board with regard to the reduction in the fees for notification of infectious diseases, the following letter (dated March 27, 1919) has been received from the Local Government Board:—

"I am directed by the President of the Local Government Board to advert to your letter of the 8th instant, regarding the fees for notifications of infectious disease and to state that Section 5 of the Local Government (Emergency Provisions) Act, 1916, which reduces the fee from 2s. 6d. to 1s., will come to an end when peace is ratified."—I am, Sir, Your Obedient Servant, (Signed) H. O. STUTCHBURY.

—I am, yours faithfully,

B. A. RICHMOND, Secretary.

The Association of Panel Committees.
Staple House, 51 Chancery Lane, W.C. 2.
March 29, 1919.

